# Utilization of Peripherally Inserted Central Catheters in Neonates Within the Neonatal Intensive Care Unit: A Decadal Single-Center Study

**DOI:** 10.7759/cureus.77904

**Published:** 2025-01-24

**Authors:** Xiangling Fu, Zhaoying Li, Lili Yao, Ijaz ul Haq, Li Wang, Liling Li, Xiaojing Hu

**Affiliations:** 1 Nursing, Children's Hospital of Fudan University, Shanghai, CHN; 2 Nursing and Nutrition, Children's Hospital of Fudan University, Shanghai, CHN

**Keywords:** catheter-related bloodstream infection, neonatal intensive care unit, neonate, peripherally inserted central catheter, single-center

## Abstract

Background: The use of peripherally inserted central catheters (PICC) in neonates has been established for numerous years, with significant advancements and enhancements observed in PICC technology over time. The objective of this study is to investigate the application trend of PICC over a 10-year period in a single-center neonatal intensive care unit (NICU).

Methodology: A retrospective analysis was conducted on the infants admitted between January 2012 and October 2021 who underwent PICC catheterization. Data pertaining to gestational age, birth weight, catheter weight, placement site, and indwelling time, as well as the occurrence of infection and complications, were collected. The data was collated, and statistical analysis was performed using IBM SPSS Statistics for Windows, Version 25.0 (Released 2017; IBM Corp., Armonk, New York, United States).

Results: Out of the 1928 enrolled infants who underwent PICC catheterization, 1033 (53.58%) were males, and 895 (46.42%) were females. On the day of placement, their mean gestational age was 30.49±0.70 weeks, and their mean weight was 1398.26±14.05 g. Over the past 10 years, the number of PICC has consistently increased. The majority of cases were due to lower and right limb catheterization, with 1749 (90.72%) lower limb cases and 1477 (76.61%) right limb cases. The duration of catheter indwelling was 19.35±0.27 days. There were 297 cases (15.40%) of catheter-related complications, which included 107 cases (5.55%) of phlebitis, 71 cases (3.68%) of catheter obstruction, and 51 cases (2.65%) of catheter-related bloodstream infection (CRBSI). The binary logistic regression analysis revealed that the catheter site (upper extremity/lower extremity) (OR=0.612; 95% CI: 0.414-0.905; p=0.014) and catheter tip position (OR=1.903; 95% CI: 1.200-3.017; p=0.006) were associated with catheter complications.

Conclusions: During neonatal PICC catheterization in the NICU, the preferred approach is to perform venous catheterization of the lower extremities and ensure that the catheter tip remains positioned within the vena cava. This technique is associated with a reduction in catheter-related complications.

## Introduction

The peripherally inserted central catheter (PICC) is typically inserted through the basilic vein, median cubital vein, and cephalic vein in the upper limb and through the great saphenous vein, small saphenous vein, or femoral vein in the lower limb. In newborns, it can also be inserted through the temporal vein of the head and posterior auricular vein. The catheter tip is usually positioned at the junction of the middle and lower third of the superior vena cava with the right atrium or at the diaphragm level of the inferior vena cava [1].

In cases of very low birth weight or critical conditions in newborns, adequate nutrition from the gastrointestinal route is often insufficient during the initial weeks of life, leading to the necessity of administering hyperosmolar or irritating medications. However, repeated intravenous catheterization can increase the risk of pain and infection and may even result in hypoxia, infection, and intracranial hemorrhage [2]. Accordingly, establishing long-term intravenous access with hyperosmolar resistance is crucial in order to rescue these critically ill neonates. Furthermore, PICC catheterization is a safe, convenient, and effective technique that is characterized by its simplicity, high success rate, minimal complications, and resistance to hypertonicity. Therefore, the application of PICC has become an indispensable part of the treatment for critically ill newborns, as it can reduce the overstimulation of the infant's blood vessels and ensure the supply of intravenous nutrition and timely administration during the rescue, thereby enhancing the infant's quality of life.

However, PICC has been associated with certain complications such as catheter blockage, catheter-related bloodstream infection (CRBSI), and ectopic catheters. Catheter blockage is a common complication during PICC catheterization and the leading cause of unplanned extubation of PICC. Studies reported that its incidence rate was 3-12.5% [3-5]. CRBSI is a prevalent and significant complication during neonatal PICC catheterization, resulting in an extension of the hospital stay, increased infant pain, and potential endangerment of the infant's life. Literature reports that the incidence of CRBSI caused by PICC placement in the neonatal intensive care unit (NICU) varies from 0.12% to 10.62% [6-8].

In this study, we examined the utilization of PICC in the NICU of the Children's Hospital of Fudan University to determine the complications and risk factors associated with its use in critical newborns.

## Materials and methods

Study design, setting, and dates 

This retrospective analysis included PICC-catheterized newborns admitted to the Children's Hospital of Fudan University NICU between January 2012 and October 2021. The NICU at the Children's Hospital of Fudan University provides care for neonates from 23 weeks of gestation to full-term. In this unit, whether the infant is born prematurely or at full-term, if the expected duration of intravenous nutrition exceeds two weeks, it is essential to insert a PICC.

Participants 

**Figure 1:**
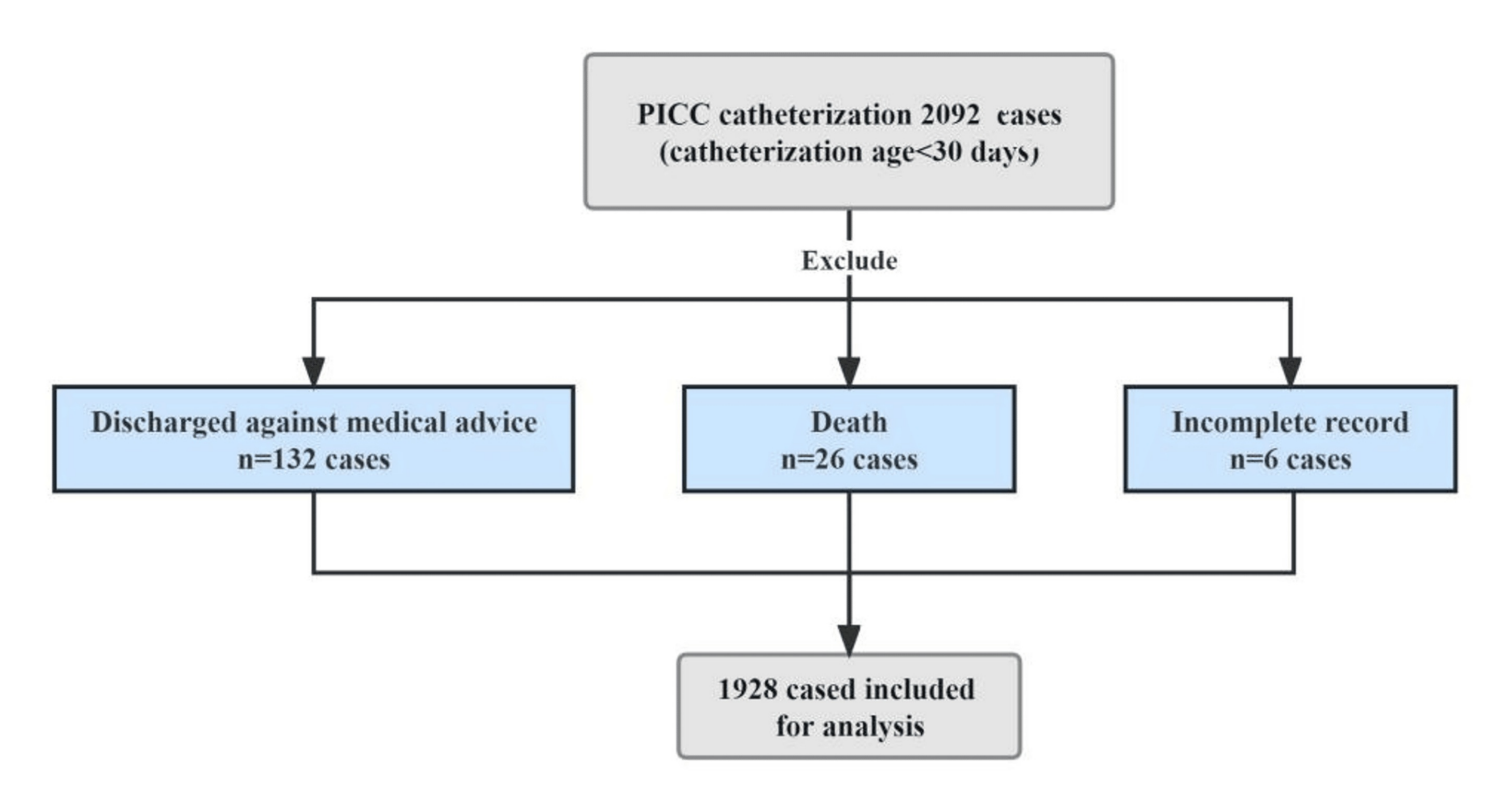
Study flowchart PICC: peripherally inserted central catheter

The inclusion criteria were infants who underwent PICC catheterization within 30 days of birth. Furthermore, if the infant had a history of multiple PICC catheterizations, they were included in the initial PICC catheterization. The exclusion criteria included infants who had severe infections (such as septicemia or necrotizing enterocolitis) prior to PICC placement, infants who died of their illness or were discharged against medical advice, and infants who did not have complete records. The study flowchart is shown in Figure 1. Among them, there were 1832 premature infants and 96 full-term infants.

PICC procedure

After obtaining the patient's signed informed consent, the nurses who qualified for PICC catheterization utilized a 1.9F single-lumen PICC (Utah, United States), and the same size of PICC was used for both term and preterm infants. The preoperative performance of routine coagulation tests was recommended. The preferred location for the placement of a PICC is the right lower limb. Prior to catheter placement, the infant's lower extremity was abducted at an angle of 45 degrees. The length of the catheter was then measured from the site of placement along the vein to the groin, umbilicus, and xiphoid process. Following this, after ensuring that the infant was maintained at a warm temperature within the isolette, comprehensive disinfection of the entire lower limb on the side of placement was conducted using an iodophor skin disinfectant. Additionally, a sterile towel was employed to establish a maximal sterile barrier. Ultrasound-guided indwelling techniques were employed for catheter placement, followed by an X-ray examination to verify the positioning of the catheter tip. The X-ray observation indicated that the catheter tip was positioned 1-2 cm outside the heart, which was regarded as an optimal location for the catheter tip. Postoperatively, a comprehensive documentation of catheter details was conducted. The information collected included the name and type of catheter, insertion vein, outcome of the procedure (success or failure), operation time, insertion depth, exposed length, circumferences of bilateral legs, as well as status of the puncture site.

PICC maintenance 

The 1.9F catheter was not intended for blood transfusion or blood drawing. Dressings were replaced as necessary, and immediate replacement was required if the dressing became curled, damaged, or contaminated. Two nurses performed the dressing change simultaneously to prevent catheter displacement or accidental dislodgement. The replacement date was documented on the dressing. The needle-free infusion connector was replaced weekly, following strict aseptic procedures. Maintenance of the catheter was carried out by nurses with PICC maintenance certification.

Definition of catheter-related complications

Phlebitis

The assessment is conducted in accordance with the diagnostic criteria for phlebitis outlined in the "2016 Standard of Practice for Transfusion Therapy" [9]. Clinical manifestations of phlebitis encompass pain/tenderness, erythema, swelling, purulence, and palpable cord-like veins.

Catheter Obstruction

Blocked flushing or an inability to flush the lumen may indicate catheter obstruction.

Survey tool and data collection methods

A custom-crafted PICC data collection table was utilized to collect the characteristics of the infants, including birth weight, gestational weeks, and other relevant details. PICC's indwelling time, catheter removal, and other related procedures were also documented. The data collection table was structured to facilitate the observation and recording of blood culture results and pathogens cultured during PICC indwelling, as well as the occurrence of catheter complications.

During the data collection process, it is the responsibility of PICC professional nurses to observe and document all processes, from catheter placement to catheter removal, for each PICC patient on the data record sheet.

Statistical analysis

The data entry and statistical analysis were performed using IBM SPSS Statistics for Windows, Version 25.0 (Released 2017; IBM Corp., Armonk, New York, United States). The measurement data, adhering to the normal distribution, were represented by the mean and standard deviation, whereas the counting data were conveyed through the number of cases and percentage. Multivariate analysis was conducted using binary logistic regression analysis. P<0.05 indicates a statistically significant difference.

Ethical approval

Approval to conduct the study was obtained from the Ethics Committee of the Children's Hospital of Fudan University (approval number: 01). The study was carried out in compliance with the Declaration of Helsinki (version in force; currently Fortaleza, Brazil, October 2013).

## Results

General information about the patients

**Figure 2:**
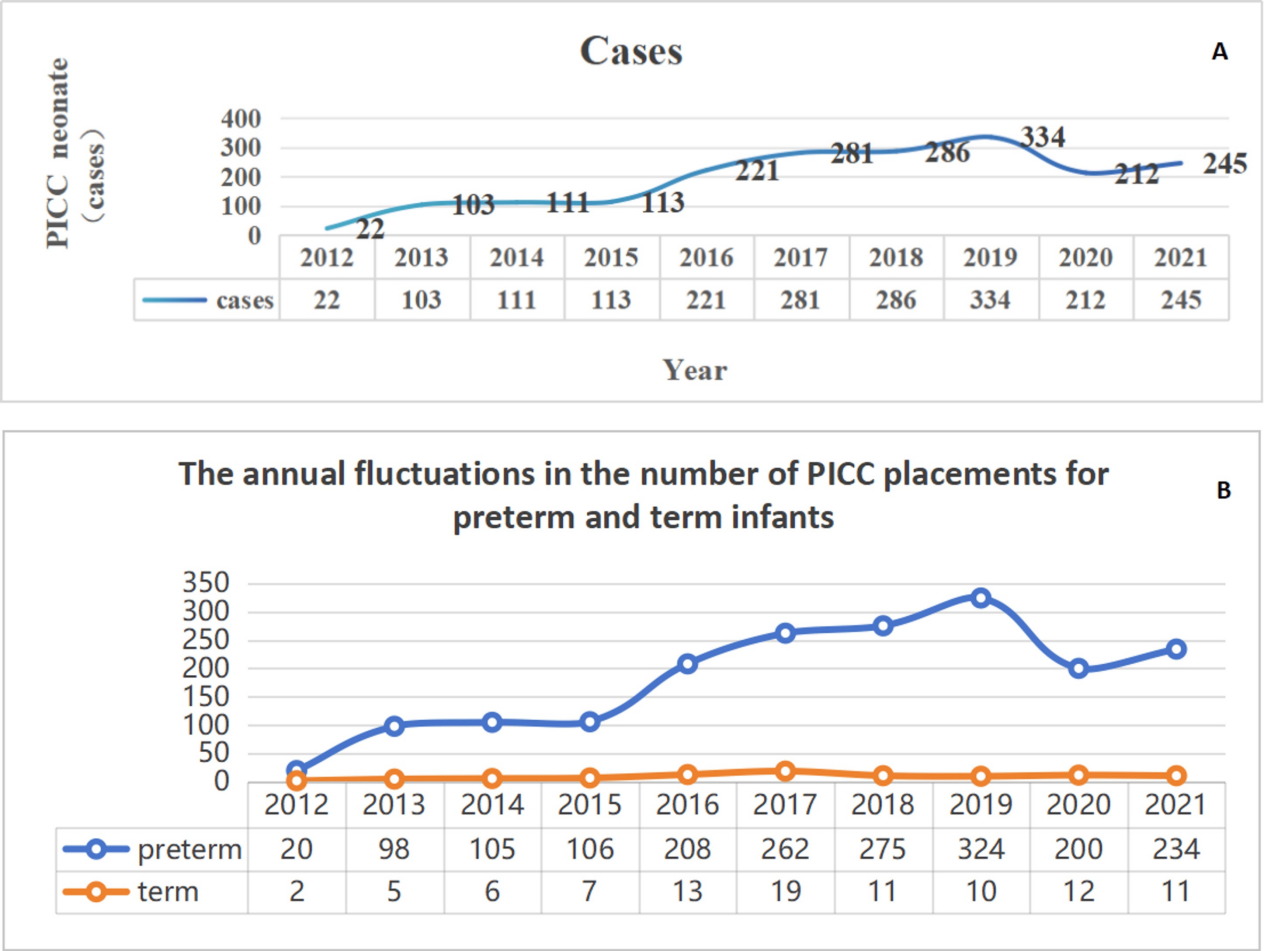
(A) Number of PICC catheterizations from 2012 to 2021 (cases/year). (B) The annual fluctuations in the number of PICC placements for preterm and term infants PICC: peripherally inserted central catheter

A total of 1,928 newborns who met the inclusion criteria were incorporated into the analysis. Preterm infants accounted for 1,832 cases, representing 95.02% of the overall population, while full-term infants comprised 96 cases, corresponding to 4.98%. The annual number of PICC placements for both preterm and full-term infants is presented in Figure 2.

**Figure 3:**
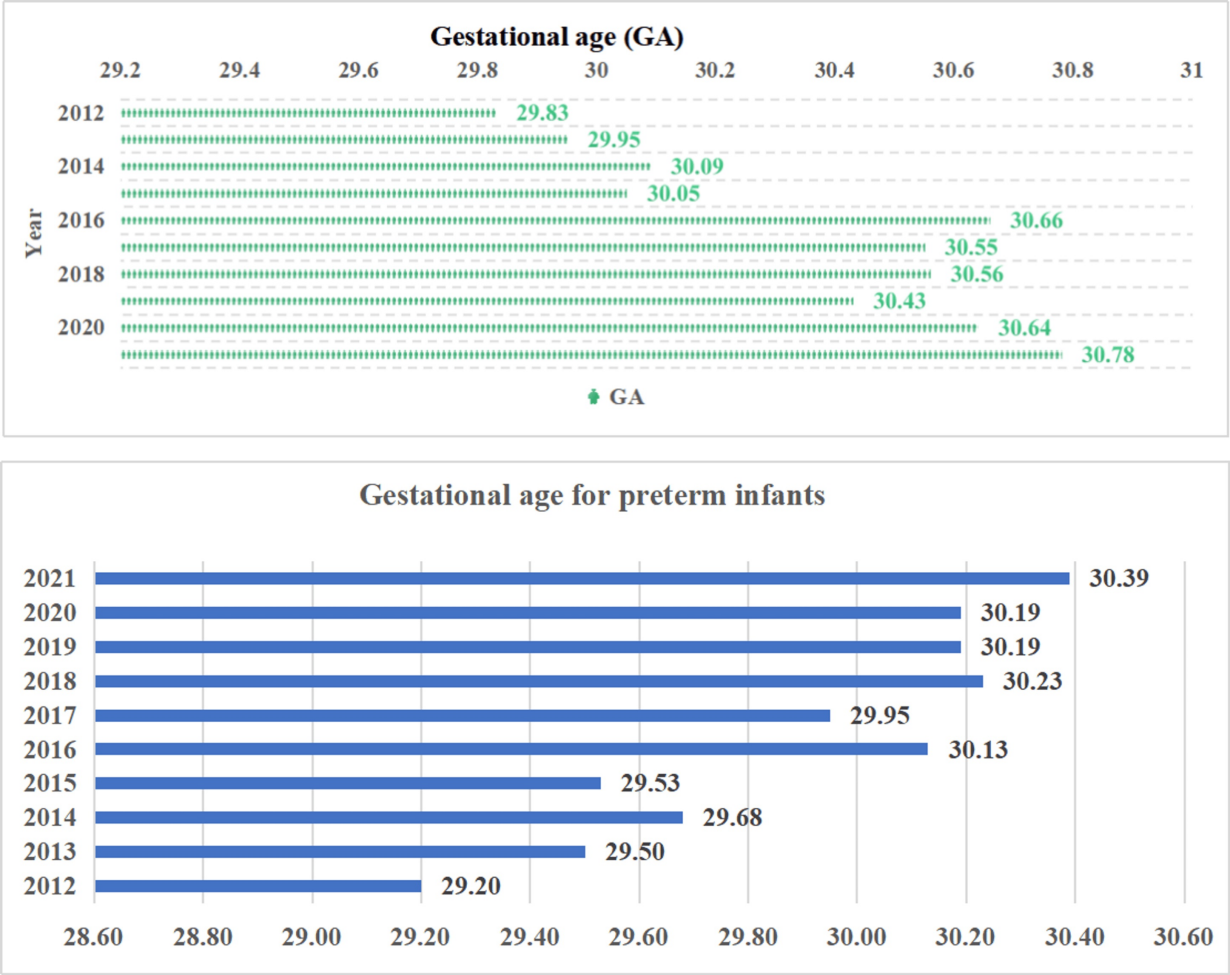
(A) Average gestational age (week/year). (B) Gestational age of preterm infants at the time of catheterization over the years

The average birth weight was 1405.68±13.38 g, while the average weight on the day of catheter placement was 1398.26±14.05 g. The mean gestational age at this time was 30.49±0.70 weeks. Figure 3 illustrates the gestational age at which preterm infants underwent catheter placement.

PICC placement sites

Table 1 displays that the primary locations for PICC placement were the lower limb and the right side, accounting for 1949 (90.7%) cases of lower limb catheterizations and 1477 (76.6%) cases of right limb catheterizations. This result may be attributed to the fact that the lower limb is our preferred site for PICC placement in this unit.

**Table 1:** PICC placement sites (n=1928) PICC: peripherally inserted central catheter

Placement sites	Cases (n)	Percentage (%)
Great saphenous vein	757	39.3
Right	619	32.1
Left	138	7.2
Femoral vein	417	21.6
Right	275	14.3
Left	142	7.4
Popliteal vein	292	15.2
Right	228	11.8
Left	64	3.3
Small saphenous vein	277	14.4
Right	223	11.6
Left	54	2.80
Basilic vein	128	6.6
Right	91	4.72
Left	37	1.9
Median cubital vein	22	1.14
Right	16	0.8
Left	6	0.3
Cephalic vein	20	1.0
Right	16	0.8
Left	4	0.21
Axillary vein	11	0.6
Right	6	0.31
Left	5	0.3
Lower extremity vein	4	0.20
Right	3	0.2
Left	1	0.1

PICC indwelling time

**Figure 4:**
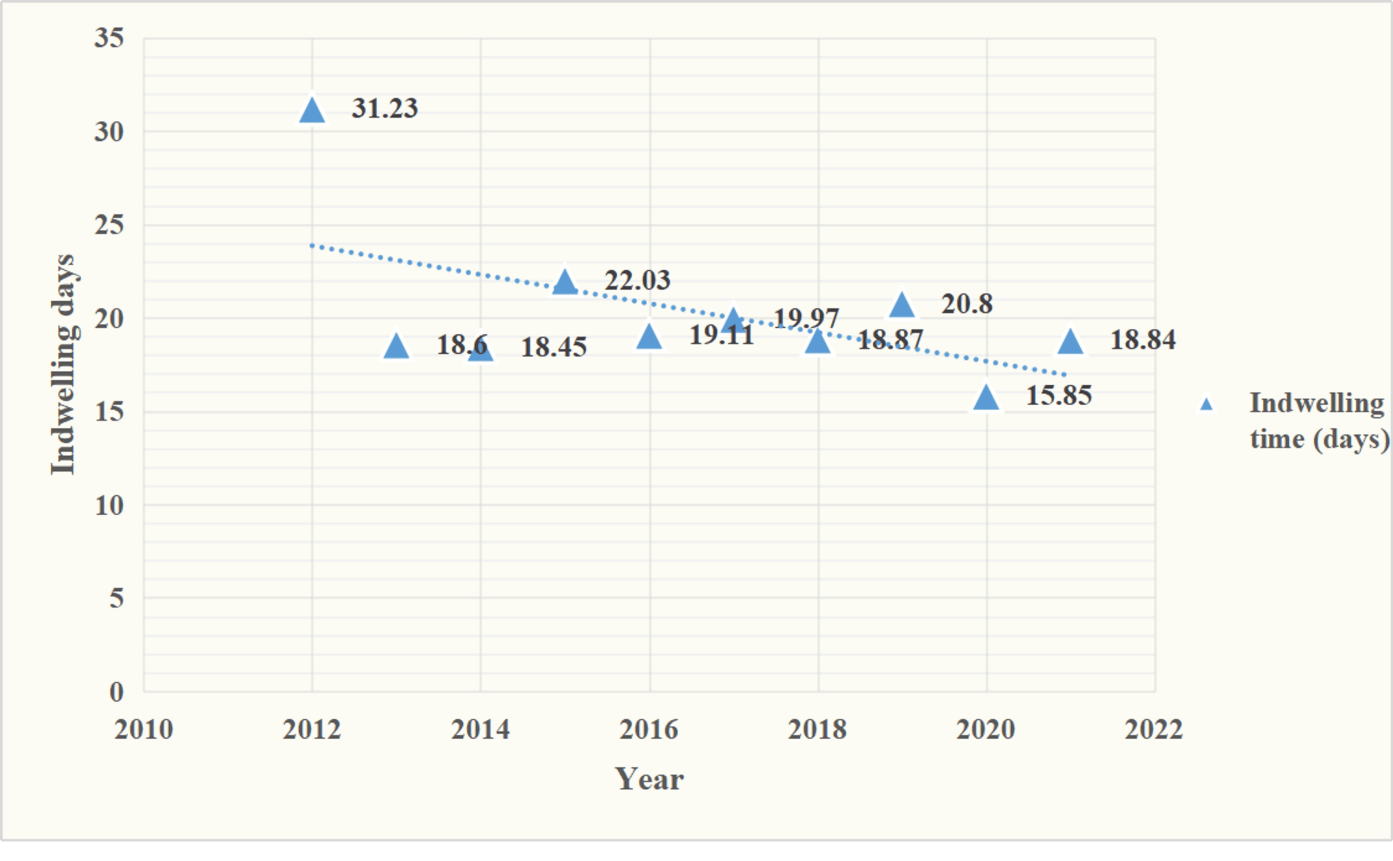
PICC average indwelling time (days/year) PICC: peripherally inserted central catheter

In the present study, the average indwelling time of the PICC was 19.35±0.27 days (range: 1-93 days), with 312 (6.2%) infants having an indwelling PICC time exceeding 30 days. Preterm infants exhibited an average duration of 19.58±0.27 days, whereas full-term infants had a shorter average duration of 14.40±1.05 days. As illustrated in Figure 4, the average indwelling catheter time generally decreased annually.

Catheter complications

**Figure 5:**
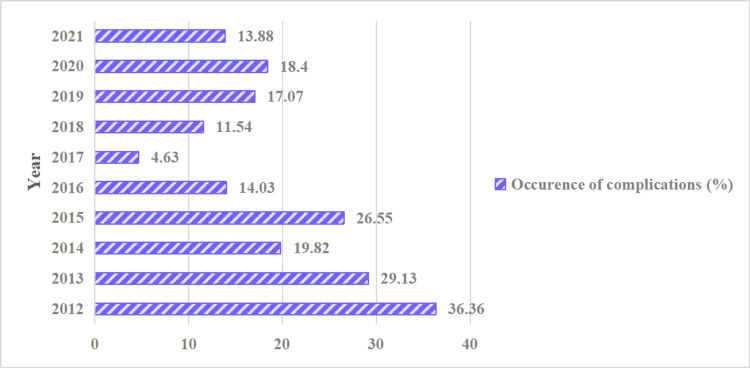
Incidence of catheter-related complications from 2012 to 2021 (%)

There were 297 (15.40%) cases of catheter-related complications. Among 1,832 premature infants, 286 cases experienced complications, resulting in an incidence rate of 15.6%. In contrast, among 96 full-term infants, there were 11 cases of complications, yielding an incidence rate of 11.5%. However, the difference between these two groups was not statistically significant (p>0.05). In Table 2, the top three items are as follows: phlebitis, observed in 107 (5.6%) cases, catheter obstruction, observed in 71 (3.7%) cases, and CRBSI, observed in 51 (2.65%) cases. Notably, all instances of phlebitis (107 cases) as well as occurrences of extravasation (four cases), pleural effusion (three cases), and thrombosis (two cases) were reported exclusively in premature infants. As illustrated in Figure 5, there has been an overall decreasing trend in the incidence of catheter-related complications over the years.

**Table 2:** PICC-related complications (n=297) PICC: peripherally inserted central catheter

Complications	Number	Percentage（%）
Phlebitis	107	5.6
Obstruction	71	3.7
Infection	51	2.7
Edema	43	2.2
Ectopic catheter	11	0.6
Extravasation	4	0.4
Catheter detachment	3	0.2
Pleural effusion	3	0.2
Thrombus	2	0.1
Difficulty in removing the catheter	2	0.1
Total	297	15.4

Correlation between catheter complications and the position of catheter insertion and tip

As demonstrated in Table 3, the binary logistic regression analysis indicates that the incidence of catheter-related complications is associated with both the placement of the catheter and the positioning of the catheter tip. The positions of catheter tips can be classified into two distinct categories. The first category encompassed 1,811 cases in which the tip was positioned within either the superior or inferior vena cava. The second category comprised 117 cases where the tip was located outside of the vena cava.

**Table 3:** Logistic regression analysis of the factors related to catheter complications in neonates with PICC PICC: peripherally inserted central catheter

Variables	B	Standard error	Wald	P	OR	95% CI
Sex	0.228	0.130	3.060	0.080	1.256	0.973-1.620
Gestational age	-0.005	0.035	0.020	0.889	0.995	0.929-1.066
Birth weight	0.000	0.000	1.610	0.205	1.000	0.999-1.000
Catheter weight	0.000	0.000	0.997	0.318	1.000	1.000-1.001
Indwelling time	-0.006	0.006	0.876	0.349	0.994	0.983-1.006
Catheter site (right/left)	-0.290	0.163	3.174	0.075	0.758	0.543-1.030
Catheter site (up/lower)	-0.491	0.199	6.056	0.014	0.612	0.414-0.905
Catheter tip position	0.643	0.235	7.487	0.006	1.903	1.200-3.017

CRBSI pathogens

Of the 1928 cases in this study, 1903 (98.7%) had their catheter tips examined. A total of 29 cases (1.50%) tested positive in catheter tip cultures. The identified bacterial species were as follows: fungi in 12 cases, *Klebsiella pneumoniae* in six cases, coagulase-negative staphylococci in four cases, *Enterobacter aerogenes* in two cases, *Escherichia coli* in one case, *Streptococcus pneumoniae* in one case, *Enterococcus faecalis* in one case, and *Staphylococcus aureus* in one case. Additionally, *Stenotrophomonas maltophilia* was detected in one case. There were a total of 170 (8.8%) positive blood culture cases identified, which included 100 instances of Gram-positive cocci, four instances of Gram-positive bacilli, 47 instances of Gram-negative bacilli, and 25 instances of fungi. Notably, among these cases, six infants exhibited simultaneous cultures of two different types of strains. Among these, a total of 51 cases (2.7%) exhibited clinical symptoms of infection and were confirmed to be CRBSI. From the positive samples, a total of 55 strains were cultured, which included 19 strains (34.6%) of Gram-positive cocci. Among these, there were 14 strains identified as coagulase-negative staphylococci, three strains classified as *Staphylococcus aureus*, and two strains recognized as *Enterococcus faecalis*. Additionally, there were 17 strains (30.9%) of Gram-negative bacilli isolated, comprising seven strains of *Klebsiella pneumoniae*, three strains of *Enterobacter cloacae*, two strains of *Enterobacter aerogenes*, and another two strains associated with xylose-oxidizing alkaligenic bacteria. Furthermore, there was also one strain each of *Escherichia coli* and *Pseudomonas aeruginosa* infection along with one strain identified as *Hafnia alvei*. Finally, a total of 19 fungi (34.6%) were also isolated from the samples analyzed. The presence of *Enterococcus* may be attributed to contamination. Additionally, the administration of antibiotics or a decline in host immunity could disrupt the symbiotic relationship between the host and *Enterococcus*, potentially resulting in infection.

## Discussion

Due to the rise in infants being born with very low birth weights, the number of NICU admissions for very low birth weight infants has steadily increased every year. Performing a puncture of peripheral veins in infants with very low birth weight is challenging due to their thin and tender skin, edema, less subcutaneous fat, small peripheral blood vessels, and shallow veins, which can easily collapse. Furthermore, the inability to tolerate repeated puncture and prolonged stimulation of drugs on peripheral blood vessels may cause extremely serious damage to very low birth weight infants, potentially resulting in hypoxia, infection, and intracranial hemorrhage in some instances [2].

The use of PICC technology in clinical practice has been on the rise due to its safety, convenience, and effectiveness. In this study, we examined the utilization trend of a PICC over a 10-year period in a single-center NICU to provide more clinical evidence for the application of PICC during hospitalization in the NICU. In the present study, of the 1928 infants who underwent PICC catheterization, 1410 (73.13%) were very low birth weight infants, and the number of PICC catheterization cases has been increasing year by year. Following the prevalence of the COVID-19 infection at the end of 2019, the number of PICC catheterization cases decreased in 2020 and 2021 as a result of fewer admitted patients. The most frequently used catheterization sites are the lower limb and the right side, particularly the great saphenous vein.

Previous studies have shown that the incidence of catheter-related complications is lower in infants with PICC catheterization in their lower limbs compared to those with catheterization in their upper limbs. According to the relevant guidelines [10], the preferred method for PICC placement in newborns is through the greater saphenous vein of the lower limb. According to reports, the one-time puncture success rate of PICC insertion through lower limb veins in neonates is high, with short procedure time, minimal bleeding, and lower total catheter-related complications compared to upper limb veins [11-13]. Furthermore, when PICC is inserted into the lower limb vein of a newborn, there exists a variation in the occurrence of complications among various insertion sites. In their study, Chen and colleagues discovered that the one-time puncture success rate, catheter blockage rate, and infection rate of PICC through the great saphenous vein were significantly better than those of PICC through the femoral vein. Furthermore, they used the evidence-based ACE Star model to evaluate PICC location in neonates and discovered that the incidence of PICC blockage was lower in the right lower limb compared to the left lower limb [12]. Therefore, the great saphenous vein of the right lower limb is the preferred option for PICC placement in clinical settings involving newborns. The average PICC indwelling time is 19.35±0.27 days, which is comparable to the median PICC indwelling time of 18 days reported by another single-center NICU in China [14] but is slightly shorter than the values reported in some previous research reports [15,16]. Reassess the necessity of maintaining a PICC in a timely manner, and promptly remove the catheter when it is no longer required for treatment or when unresolved complications arise. There was one infant observed who had a prolonged indwelling of a neonatal PICC exceeding 50 days, which presented challenges during removal. This situation prompted us to consider the necessity of removing the catheter as early as possible.

The present study discovered that the incidence of complications associated with PICC catheterization in NICU infants has decreased. In 2016, the unit underwent a continuous quality improvement project focused on PICC, which led to a significant reduction in catheter-related complications in 2017 following the implementation of a series of cluster strategies. During the quality improvement project's curing period in 2018-2019, there was a notable increase in the incidence of catheter-related complications. Taking this fact into account, the unit implemented a quality improvement strategy for PICC in 2020, leading to a significant enhancement. The main components of quality improvement include the advanced training of the PICC professional team, the implementation of a standardized catheterization method based on guidelines, the selection of placement sites, the maximization of sterile barrier, the selection of disinfectants and disinfection methods, the enforcement of hand hygiene, the timing of dressing replacement, the disinfection method of the infusion interface, the frequency of interrupting the infusion system, the flushing method, the utilization of low-dose antifungal medications (our center recommends administering fluconazole at a prophylactic low dosage of 3 mg/kg every 48-72 hours), and daily monitoring of the catheter, as well as the timing of catheter removal and other related aspects [9,17,18]. 

Our findings revealed that the total incidence of catheter-related complications of lower limb catheterization was lower than that of upper limb catheterization and the difference was statistically significant, which is consistent with previous literature [11-13]. It is recommended that priority be given to the lower limb for catheterization when performing PICC placement in newborns to minimize complications. The catheter tip should be positioned after PICC insertion. The current study indicated that the rate of catheter-related complications was comparatively low when the catheter tip was positioned in the central vein, which aligns with certain prior research findings [19,20].

According to the PICC practice guide for newborns, the optimal placement for a PICC in this population is in the vena cava [21]. Meanwhile, the 2016 version of the "Practice Standard for Infusion Treatment" by the Infusion Nurses Society (INS) [9] suggests that the best location for the PICC tip for upper limb catheterization is the junction point of the superior vena cava and the right atrium. For lower limb catheterization, the tip of the PICC should be positioned in the inferior vena cava above the diaphragm level. Additionally, it is important to note that optimal performance of the procedure is essential for this tip position. The current study revealed that 297 out of 1928 neonates who underwent PICC catheterization experienced catheter-related complications, resulting in an incidence rate of 15.40%, which is slightly higher than the previously reported incidence of neonatal PICC catheterization complications, which ranges between 9.3% and 10.71% [16,22]. It may be due to our routine practice of not changing the catheter and the slightly prolonged indwelling time.

The primary complications, consistent with literature reports, were phlebitis and catheter obstruction, observed in 5.55% and 3.68% of cases, respectively [22,23]. Phlebitis may be associated with a too-thin vessel, the material of the PICC, the insertion tools used, and the speed of catheter insertion [24-26]. In this center, we do not utilize heparin to prevent occlusion. The occlusion of the catheter may be attributed to the design of the 1.9F catheter being too delicate, as newborns typically use this catheter. Furthermore, the catheter occlusion may also be caused by the premature infants' hypercoagulable state, improper operation and maintenance by operators, too slow or sudden interruption of infusion speed, non-positive pressure sealing after infusion, or incorrect sealing techniques [24,27].

In the present study, it was found that out of 1928 neonates who underwent PICC catheterization, 51 experienced CRBSI during the procedure. Furthermore, the occurrence of CRBSI was 2.67%, with the pathogenic bacteria predominantly being Gram-positive cocci (34.55%), fungi (34.55%), and Gram-negative bacteria (30.91%). A majority of literature reports from developed countries indicate that Gram-positive bacteria constitute more than half of the pathogenic bacteria causing CRBSI, whereas fungi account for a relatively lower proportion [28,29]. The proportion of fungi and Gram-negative bacteria in this study was equivalent to that of Gram-positive cocci, which is significantly distinct from the spectrum of CRBSI pathogenic bacteria prevalent in developed countries. According to studies conducted in China and certain developing countries, the pathogenic bacteria responsible for CRBSI primarily belong to the Gram-negative bacterial species [30,31]. The results of this study are consistent with those of the majority of developing countries. The relatively poor medical and health conditions in developing countries may account for the higher presence of Gram-negative bacteria in their CRBSI pathogens. The majority of infants admitted to the NICU are those who are born with very low birth weight and are unable to receive adequate nutrition from their gastrointestinal tract within a short period of time after birth. As a result, they require long-term intravenous parenteral nutrition as a means of life support. Research suggests that fungal infections may be induced by broad-spectrum antibiotics and prolonged intravenous nutrition [32,33]. Furthermore, it has been discovered that the presence of fungi in the pathogenic agents of CRBSI is significantly elevated with an increase in the number of days of catheterization [16]. Consequently, there is an urgent need to enhance antibiotic management strategies. Additionally, greater emphasis should be placed on the early initiation of complete enteral nutrition, even if trophic feeds have commenced on the first day post-birth.

There were several limitations of the study. This retrospective analysis of PICC catheterization in a single-center NICU may limit the generalizability of the results. Another limitation of the study was the lack of a control group or comparison group. This study primarily described the application of PICC in NICU newborns; however, it did not establish a control group or comparison group to evaluate the advantages and disadvantages of various PICC management methods. Future research should consider designing prospective studies that incorporate groups to compare the benefits and drawbacks of different management approaches.

## Conclusions

The proportion of PICC applications in the NICU has significantly increased, particularly among infants with very low birth weight. The gestational age of the infant was relatively low when the catheter was placed; however, the incidence of complications has decreased significantly. The incidence of complications related to PICC was closely linked to the placement of the catheterization and the positioning of the catheter tip within the central vein. To ensure that the catheter tip is positioned at the vena cava, it is recommended to first place the PICC in the veins of the lower and right limbs. A high proportion of CRBSI strains were caused by fungi and Gram-negative bacteria. Thus, it is imperative to enhance the management of neonatal antibiotics in the NICU, ensure the prompt initiation of enteral nutrition, minimize the duration of PICC use, and decrease the frequency of CRBSI.
